# Refining medical clearance protocol for patients with primary psychiatric complaints in the emergency department

**DOI:** 10.3389/fpsyt.2023.1209450

**Published:** 2023-07-10

**Authors:** Christopher Austin Casey, Jaime Guzman, Mckailey Salard, Natalie Wu, Ross Rieger, Payton Mangham, James Patterson

**Affiliations:** Louisiana State University Health Shreveport, Shreveport, LA, United States

**Keywords:** ED labs, CK, medical clearance, psychiatry, ED clearance

## Abstract

**Introduction:**

Medical clearance for patients with primary psychiatric complaints presenting to the emergency department has been debated for decades. Emergency physicians have argued that clearance labs are unnecessary, yet psychiatrists may still order or require them. A retrospective review was conducted to evaluate the continued need for labs of psychiatric patients and help identify high risk groups that may need additional intervention prior to medical clearance.

**Methods:**

Charts of 163 patients from Ochsner LSU Shreveport Psychiatric Crisis Unit (PCU) were reviewed with data collected of history, physical examination, review of systems, vitals and routine lab work including complete blood count (CBC), comprehensive metabolic panel (CMP), urine drug screen (UDS), serum ethanol level (EtOH), urinalysis (UA), creatine kinase (CK), urine pregnancy test (UPT), and rapid COVID-19.

**Results:**

Review identified 82 patients (50.3%) that received interventions prior to medical clearance. Most common intervention was intravenous (IV) fluids (*n* = 59; 45%) followed by admission to other service (*n* = 15; 8.4%), imaging (*n* = 10; 7.6%), antihypertensive medication (*n* = 3; 3.1%), cardiac workup (*n* = 3; 2.3%), antibiotics (*n* = 3; 2.3%), lorazepam for undocumented reasons (*n* = 2; 1.5%). Additional interventions completed once included immunizations, antiseizure medication, pain medication, and additional lab work. Causes for IV fluids were reviewed with elevated creatine kinase (CK) (*n* = 31; 50.8%) being most common. Additional causes included undocumented (*n* = 12; 19.7%), tachycardia (*n* = 6; 9.8%), elevated EtOH level (*n* = 3; 4.9%), dehydration (*n* = 2; 3.3%), acute kidney injury (AKI) (*n* = 2; 3.3%), leukocytosis following a seizure (*n* = 1; 1.6%), elevated CK and leukocytosis (*n* = 1; 1.6%), and AKI and elevated CK (*n* = 1; 1.6%). Most common cause for medical admission was elevated CK being cited in 8 out of 15 admissions (53.3%). Additional causes for admission included AKI (*n* = 2; 14.3%), seizures and leukocytosis (*n* = 1; 6.7%), rule out of acute coronary syndrome (ACS) (*n* = 1; 6.7%), alcohol withdrawal (*n* = 1; 6.7%), encephalopathy with drop in hemoglobin and white blood cell count (*n* = 1; 6.7%), and encephalopathy with elevated CK (*n* = 1; 6.7%).

**Discussion:**

Our results support the recommended guidelines set by AAEP for laboratory testing in addition to history, vital signs and physical examination prior to medical clearance. Certain laboratory testing such as CK and CMP were identified to have higher utility for medical intervention while other lab work such as UA and UDS had less of an impact. Further, we suggest that specifically a CK and CMP be obtained on patients presenting with any of the following: agitation, abnormal vital signs, intoxication, or a history of or current stimulant use as these were factors correlated with lab abnormalities that led to interventions.

## Introduction

Inpatient psychiatric facilities often have limited capabilities to treat emergent or even urgent medical problems, and while some facilities have a hospitalist on staff to evaluate and treat medical needs, others do not. Additionally, some patients have medical causes for their psychiatric symptoms that require admission to other services besides psychiatry. Therefore, psychiatric facilities usually rely on the referral source to exclude serious medical causes, address medical issues that either require urgent or emergent treatment, and to ensure that patients are appropriate for admission. This process is typically called medical clearance.

Mandatory routine labs have been a part of the clearance process for decades (1–3). One assertion that has been used in the past as justification for routine screening labs is that psychiatric patients do not reliably or accurately report symptoms to guide testing. However, one study found that the initial medical complaints correlated directly with the need for medical clearance (4). Other studies have indicated that those with normal physical exams, stable vital signs, and no physical complaints do not require laboratory testing (4, 5).

The American Association for Emergency Psychiatry published guidelines in 2017 with eight recommendations for the process of medical clearance of adult psychiatric patients. They recommended universal screening involving, at a minimum, vital signs, history, physical examination, and assessment of mentation. They recommended that the decision for further evaluation be based on the emergency physician’s assessment. They also identified further areas requiring investigation, one of which was the question of which criteria would define groups at high risk for medical disease (6).

At our institution, standard labs without clinical indication are required in the emergency department primarily because most receiving facilities do not accept patients without them. This retrospective chart review was conducted to evaluate the need for the routine labs on our panel. More specifically we evaluated their usefulness based on whether they directly led to interventions and whether they affected final disposition. Secondarily, this study was conducted to attempt to separate high-risk groups requiring further laboratory testing from low-risk groups who may not require labs.

## Materials and methods

This retrospective chart review included psychiatric patients screened in our ED for admission to inpatient psychiatric facilities between 3/2/2021 and 9/1/2021. Patients were included if they presented to the ED with a primary complaint that was psychiatric in nature. Patients were excluded if they: did not have symptoms that required admission, were prisoners, were employees of the hospital, expired in the ED, were younger than 18, or did not have all routine labs obtained.

We reviewed 300 charts meeting inclusion criteria. Of those, 163 also met exclusion criteria. At our institution, a psychiatric crisis unit (PCU) exists where patients are admitted after medical clearance in the ED but prior to transfer to inpatient units. Admission to the PCU served as a proxy for admission to an inpatient unit since all patients admitted to the PCU required routine labs and because all transfers to inpatient units occurred after admission to the PCU. Charts were reviewed by accessing ED notes written by emergency department residents and psychiatry residents working in the PCU and by reviewing lab results.

Data collected from the notes included HPI, review of systems (ROS), physical examination, vital signs, and assessment and plan. ROS was recorded as negative if no systems were positive besides psychiatric. However, within the psychiatric ROS, we recorded agitation as positive due to the well-known requirement for intervention at times by ED staff in the form of chemical sedation. The physical exam was marked negative if no findings were documented and if the initial blood pressure, pulse rate, respiratory rate, and oxygen saturation were within normal limits. The blood pressure range considered normal in this study was 90–139 systolic and 60–79 diastolic. The pulse rate range considered normal was 60–100. The respiratory rate range considered normal was 12–20. The oxygen saturation cutoff for normal was 95%. The specific physical exam findings documented were recorded.

Labs included in the routine testing panel were complete blood count (CBC), complete metabolic panel (CMP), urinalysis (UA), urine pregnancy test (UPT), urine drug screen (UDS), serum EtOH, serum creatine kinase (CK), and rapid COVID-19. The specific levels or CK and EtOH were recorded. For the CBC, CMP, UA, UPT, and COVID-19, results were recorded simply as either normal or abnormal. For the UDS, the specific substance or substances that were detected were recorded. We recorded the final disposition in the ED and PCU notes as well as any interventions performed by the ED and PCU residents.

## Results

The demographic data of the patient population is presented below:

**Table:** 

Demographics	
Age	Mean = 36
	Median = 33
	Range = 18–72
Sex	M = 67%
	F = 33%

Of the 163 patients meeting inclusion and exclusion criteria, 82 patients (50.3%) received interventions, and a total of 105 interventions were performed by the ED. The interventions performed were as follows in descending order of frequency: intravenous (IV) fluids (*n* = 59; 45.0% of all interventions), admission to other services (*n* = 15; 11.5%), potassium replacement (*n* = 13; 9.9%), PRN medications for agitation (*n* = 11; 8.4%), imaging (*n* = 10; 7.6%), antihypertensive medication for high blood pressure (*n* = 4; 3.1%), cardiac workup (*n* = 3; 2.3%), antibiotics (*n* = 3; 2.3%), consults to other services (*n* = 3; 2.3%), and lorazepam for undocumented reasons (*n* = 2; 1.5%). Other interventions that were only performed once included acetaminophen administration for pain, folic acid and thiamine administration for alcohol use, laceration repair, tetanus immunization for a wound, repeat hemoglobin level, chlordiazepoxide administration for elevated alcohol level, thyroid panel for a history of thyroid nodule, and levetiracetam administration for seizure.

**Table:** 

Interventions performed in the ED	
**Intervention**	**Number of patients**
IV fluids	59
Admission to other services	15
Potassium replacement	13
PRN medications for agitation	11
Imaging	10
Antihypertensives	4
Cardiac workup	3
Antibiotics	3
Consultation to other services	3
Lorazepam	2
Acetaminophen	1
Tetanus immunization	1
Repeat hemoglobin level	1
Chlordiazepoxide	1
Thyroid panel	1
Levetiracetam	1

IV fluids were given for the following documented reasons in descending order of frequency: elevated CK (*n* = 31; 50.8% of all IV fluids), undocumented (*n* = 12; 19.7%), tachycardia (*n* = 6; 9.8%), elevated EtOH level (*n* = 3; 4.9%), dehydration (*n* = 2; 3.3%), acute kidney injury (AKI) (*n* = 2; 3.3%), leukocytosis following a seizure (*n* = 1; 1.6%), elevated CK and leukocytosis (*n* = 1; 1.6%), and AKI and elevated CK (*n* = 1; 1.6%). Elevated CK alone was also the most cited sole factor for all interventions at 29.5%.


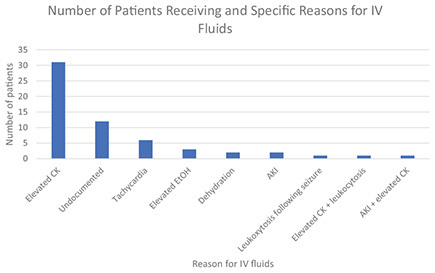


The commonest reason for admission to another service was elevated CK, which was cited for 8 of out the 15 admissions (53.3%). Patients were also admitted for AKI (*n* = 2; 14.3%), seizures and leukocytosis (*n* = 1; 6.7%), rule out of acute coronary syndrome (ACS) (*n* = 1; 6.7%), alcohol withdrawal (*n* = 1; 6.7%), encephalopathy with drop in hemoglobin and white blood cell count (*n* = 1; 6.7%), and encephalopathy with elevated CK (*n* = 1; 6.7%).


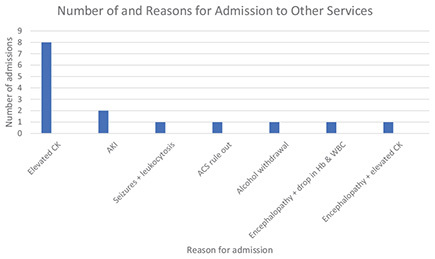


Imaging was obtained for 3 patients due to pain, for one due to cough, and for one due to an undocumented reason. A head CT was obtained for new onset psychosis for 4 patients and for a wound for one patient.

Antibiotics were given to one patient for cellulitis. They were given to 2 other patients for urinary tract infections, both of which were asymptomatic.

Consults to neurology, oral and maxillofacial surgery, and ophthalmology were placed for seizures, fracture, and photophobia, respectively.

One patient had an abnormal hemoglobin level repeated, which was normal.

## Discussion

The preferred method of medical clearance in the ED has been debated (5, 7). In some EDs, a specific set of labs is ordered as a screening panel regardless of findings on history and physical exam (2, 5, 7). These labs can be costly. For example, one study published in 2012 found that when the psychiatry service ordered laboratory or radiographic studies on 191 patients for whom emergency physicians did not order ancillary testing for medical clearance, the monetary impact based on Medicare reimbursement rates was $37,682 (2). Of those 191 patients, only one patient’s disposition was altered. Another major reason why they continue to be required is that some psychiatric facilities have limited medical resources and therefore rely on EDs to obtain labs prior to admission. On the other hand, previous research and guidelines have indicated that routine labs on all psychiatric patients are not needed. Our results support the AAEP guideline recommending laboratory testing be guided by history, physical exam, and vital signs.

Our results highlight the need for fluids in a surprising number of patients. They were by far the commonest intervention. The primary documented reasons for fluids were elevated CK levels, elevated EtOH levels, CMP results, and vital signs, although for 19.7% of those receiving fluids there was no specific documented reason. Dehydration was cited as a reason, and in one case the patient was tachycardic and disoriented. The other patient was agitated. In two cases, leukocytosis was cited as one, but not the only, factor. In all cases except one, at least one of the following findings on history and physical was present: elevated blood pressure, tachycardia, incomplete ROS due to mental status, substance abuse reported by the patient, history of substance abuse, intoxication, agitation, violence, and exercising reported by a psychotic patient. The lone case for which none of these was found on history and physical examination also had no documented reason for IV fluids. The patient was a 23-year-old homeless male but had no history of drug use, did not report drug use, and had normal vital signs. IV fluids were given prior to labs drawn, and the patient’s labs, once drawn, were unremarkable. Possibly, homelessness could be a component of the history that suggests a need for further lab evaluation or IV fluids.

CMP results led to both interventions and admissions. AKIs in our study were associated with agitation, tachycardia, elevated blood pressure, or a history of or current amphetamine use in all cases. For two patients, fluids were given in the ED solely for AKIs and partially for an AKI in another case. These patients were admitted to the PCU. Two others were admitted to internal medicine solely for AKIs. Of the 13 patients receiving potassium replacement, each had at least one of the following findings: tachycardia, elevated blood pressure, history of substance abuse, intoxication, or agitation. Therefore, a BMP or CMP should be considered in patients who have agitation, intoxication, abnormal vital signs, a history of amphetamine use, or current amphetamine use reported by the patient. In addition, since all of these patients for which interventions were performed had at least one of the above findings, the CMP appears better utilized as a lab directed by specific findings rather than as part of a general screening process for all psychiatric patients.

CK results alone led directly to both the most interventions performed in the ED (23.6%) and the most admissions (53.8%), and it was cited as part of the reason for interventions and admissions in still more cases. In all cases (100%) involving either CK or AKI, one of the following abnormalities was present on history and physical examination: agitation, tachycardia, elevated blood pressure, and either a history of amphetamine use or current amphetamine use reported by the patient. Therefore, our results suggest that patients with agitation, tachycardia, elevated blood pressure, a history of amphetamine use, or current amphetamine use reported by the patient should have a CK level and at least a basic metabolic panel (BMP), if not CMP, drawn. The results also suggest that the decision to obtain CK levels could be guided by the above findings rather than as part of a routine lab panel for all patients.

While in our study only amphetamines were associated with AKI and elevated CK levels, other stimulants have also been associated with elevated CK levels, rhabdomyolysis, and AKIs (8–11). Therefore, it seems reasonable to obtain CK levels and a BMP or CMP in these patients.

As for the utility of the CBC, in only one out of 163 cases was it cited as the only lab as a reason for intervention. In this case, the hemoglobin was low, but the CBC was repeated and the hemoglobin returned normal. The CBC was cited as one of the reasons for fluids in two cases. In one, fluids were given for leukocytosis and a seizure. In this case, laboratory testing including a CBC would have been indicated based on the seizure. In the other case, the other factor cited was elevated CK. In none of these cases was the disposition affected. It was cited as part of the reason for admission in one case of encephalopathy. However, encephalopathic findings on physical exam would typically necessitate collection of CBC, and this patient also had a history of HIV. Therefore, our results do not support the use of the CBC as part of routine labs for screening of all psychiatric patients.

The remaining patients admitted had clear indications for lab testing. The patient admitted for alcohol withdrawal was symptomatic. The patient admitted for ACS rule out had chest pain and shortness of breath.

The UPT was negative in all cases. However, this is required by most if not all facilities as they either do not accept pregnant patients or only accept patients whose pregnancies have not progressed beyond a certain stage. This is for safety. Therefore, a UPT will still be required as part of the screening process.

UA results led to treatment with oral antibiotics in some cases, but it did not change the disposition in any case. Therefore, its utility in general medical clearance, *per se*, based solely on our results is low since oral antibiotics can be started or continued on inpatient units. However, given the association of urinary tract infections and delirium in the elderly (>65 years), a urinalysis seems a reasonable component of the general screening process in the geriatric population (12).

The COVID-19 test was negative in all cases. However, it still seems a reasonable part of the screening process as of today because this will determine whether a patient requires admission to a dedicated COVID unit at an inpatient psychiatric facility. Most facilities do not have COVID units and therefore could not accommodate these patients.

The EtOH level alone was cited as the reason for fluids for 3 patients. This did not affect the disposition for any of these patients. One of these patients was tachycardic and hypertensive. The other two patients were hypertensive and agitated. Thus, all these patients had components of their physical exam and vital signs that could have been used to direct diagnostic testing, including an EtOH level.

UDS results were not cited as the reason for any intervention. This suggests that its role as part of the screening process is limited. Our results are consistent with a literature review that found the UDS was unlikely to affect management in the ED (13).

15 patients were admitted to other services. 10 were admitted to medicine services solely due to elevated CK, and each of them had agitation, abnormal vital signs, or amphetamine use reported by the patient. The remaining 5 patients admitted had clear indications for lab testing. The patient admitted for alcohol withdrawal was symptomatic. The patient admitted for ACS rule out had chest pain and shortness of breath. Two patients were admitted for encephalopathy, and the last was admitted for seizures. Therefore, these findings suggest labs be used as part of a diagnostic process guided by history and physical exam rather than as part of a general screening of all psychiatric patients.

## Limitations

As our hospital is a residency training hospital, the level of training among residents varied. In addition, a psychiatry resident evaluated each patient after evaluation by an ED resident, and many EDs do not have a psychiatrist on site to evaluate patients prior to transfer for admission. Another limitation concerns the closeness of the relationship between our psychiatric emergency area and the main ED. Patients who are medically cleared are quickly and easily sent to the PCU, and patients initially cleared but inappropriately so can easily be transferred back to the main ED. This sometimes leads to a hastier clearance process in our ED, whereas at other EDs that do not have an emergency psychiatric service this may not be the case. In our experience with psychiatric hospitals in the state, some are unwilling to accept patients with certain lab values that our PCU is willing to accept. For example, some do not accept patients whose CK levels are above 500 U/L, whereas our PCU allows CK levels sometimes above 1,000 U/L depending on the psychiatry resident at the time. This indicates that the number of interventions in our ED for elevated CK levels may be lower at our hospital than at others. Finally, our study was limited to a single site and used a small sample size.

## Conclusion

In their 2017 consensus recommendations, the AAEP outlined the need to define groups at high risk for medical disease (6). Our results further delineate certain groups and the specific laboratory tests that should be obtained. We suggest that a CK level, EtOH level, and a CMP or BMP be considered as part of the diagnostic process for patients presenting with agitation, abnormal vital signs, intoxication, or a history of or current stimulant use. Patients with at least one of these elements required interventions including IV fluids in the ED and admission to other services. The UDS had the least utility in the medical clearance process as it was not cited as the reason for any intervention or admission, and our results are consistent with previous research indicating low utility in the clearance process (6, 14). However, it does direct treatment planning for subsequent care, and the AAEP recommendations advised similarly (6). Inpatient units can obtain a UDS, but it is best utilized for this purpose as soon as possible on arrival to the ED due to rapid metabolism of certain illicit substances. Therefore, while not necessary for medical clearance, *per se*, it still has value in the ED. The CBC was also of low utility, consistent with previous research (15). UA results led to oral antibiotic treatment in some cases. However, since no patient was admitted to another service and since oral antibiotics can be started or continued on inpatient units, the UA appears to be of low utility in the general medical clearance process. It may be better utilized in a more directed manner, such as in elderly patients to screen for delirium (12). Although COVID-19 results and the UPT were negative in all cases, these tests are still required as most inpatient units are not equipped for patients testing positive for these.

## Data availability statement

The raw data supporting the conclusions of this article will be made available by the authors, without undue reservation.

## Ethics statement

The studies involving human participants were reviewed and approved by Matthew Vetkoetter Louisiana State University Health Shreveport. Written informed consent for participation was not required for this study in accordance with the national legislation and the institutional requirements.

## Author contributions

CC wrote the primary manuscript, developed the study, and performed chart reviews. JG wrote the abstract. MS, NW, PM, and RR performed chart reviews. JP supervised the study. All authors contributed to the article and approved the submitted version.
